# Profiling lifetime episodes of upper gastrointestinal bleeding among patients from rural Sub-Saharan Africa where schistosoma mansoni is endemic

**DOI:** 10.11604/pamj.2016.24.296.9755

**Published:** 2016-08-03

**Authors:** Christopher Kenneth Opio, Francis Kazibwe, Ponsiano Ocama, Lalitha Rejani, Elena Nikolaevna Belousova, Paul Ajal

**Affiliations:** 1 Makerere University, College of Health Sciences, P.O.Box 7072, Kampala, Uganda; 2 Bishop Stuart University, Public Health Department, P.O.Box 9, Mbarara, Uganda; 3 Kazan University, Russian Federation; 4 Pakwach Health Centre IV, Ministry of Health, Pakwach, Uganda

**Keywords:** Upper gastrointestinal bleeding, Sub-Saharan Africa, hepatic schistososomiasis

## Abstract

**Introduction:**

Severe chronic hepatic schistosomiasis is a common cause of episodes upper gastrointestinal bleeding (UGIB) in sub-Saharan Africa (SSA). However, there is paucity of data on clinical epidemiology of episodes of UGIB from rural Africa despite on going public health interventions to control and eliminate schistosomiasis.

**Methods:**

Through a cross sectional study we profiled lifetime episodes of upper gastrointestinal bleeding and associated factors at a rural primary health facility in sub-Saharan Africa were schistosomiasis is endemic. The main outcome was number of lifetime episodes of UGIB analyzed as count data.

**Results:**

From 107 enrolled participants, 323 lifetime episodes of UGIB were reported. Fifty-seven percent experienced ≥ 2 lifetime episodes of UGIB. Ninety-four percent had severe chronic hepatic schistosomiasis and 80% esophageal varices. Alcohol use and viral hepatitis was infrequent. Eighty-eight percent were previously treated with praziquantel and 70% had a history of blood transfusion. No patient had ever had an endoscopy or treatment for prevention of recurrent variceal bleeding. Multivariable analysis identified a cluster of eight clinical factor variables (age ≥ 40, female sex, history of blood transfusion, abdominal collaterals, esophageal varices, pattern x periportal fibrosis, anemia, and thrombocytopenia) significantly associated (P-value < 0.05) with increased probability of experiencing two or more lifetime episodes of UGIB in our study.

**Conclusion:**

Upper gastrointestinal bleeding is a common health problem in this part of rural SSA where schistosomiasis is endemic. The clinical profile described is unique and is important for improved case management, and for future research.

## Introduction

Upper gastrointestinal bleeding (UGIB) is a common worldwide health problem associated with substantial health care cost, morbidity, and death [1–4]. Patients with UGIB may experience one or more lifetime episodes of UGIB. Each new episode of UGIB increases one’s risk of morbidity and death. Among the causes of upper gastrointestinal bleeding, variceal upper gastrointestinal bleeding or UGIB due malignancy have the highest mortality [5]. Variceal upper gastrointestinal bleeding, a complication of portal hypertension is the most frequent type of upper gastrointestinal bleeding in sub Saharan Africa [6–9]. The usual causes of portal hypertension include schistosomiasis mansoni (through periportal fibrosis) and liver cirrhosis (due to chronic viral hepatitis, alcoholic liver disease, and others) [10–13]. In the absence of treatment up to 50% with severe chronic hepatic schistosomiasis of Mansoni type and/or chronic viral hepatitis will develop esophageal varices and more than half will experience upper gastrointestinal bleeding during their lifetime. In liver cirrhosis, 4 of 10 that develop acute variceal UGIB will die at 6 weeks, one-third re-bleed thereafter, and only one-third survive beyond 1 year [11, 14]. The outcome is much better in UGIB due to schistosomiasis. In this group, 50 to 70% of those who experience their first episode of UGIB will go on to experience more episodes of bleeding over 5 years and one-third will die as a result of bleeding during the same period [15–20]. However, treatment of varices with drugs, bands, and shunts is shown to prevent further episodes of UGIB [21–23]. On the other hand, there is paucity of data on clinical epidemiology of lifetime episodes of upper gastrointestinal bleeding from rural Africa where schistosomiasis is endemic. We studied patients with one or more episodes of UGIB and determined the factors associated with number of lifetime events among patients presenting at a rural primary health facility in sub Saharan Africa. Our specific study objective was to determine the clinical, laboratory and ultrasound findings associated with increased probability of experiencing two or more episodes of UGIB is this part of rural SSA.

## Methods

### Ethics statement

This was routine cross sectional study that involved human participants. It was approved by School of Medicine, Makerere University, Institutional review board, Kampala, Uganda (#REC REF2011-244), and the Uganda National Council for Science and Technology, Kampala, Uganda (UNCST approval #, HS 1620). The study was conducted according to the principles expressed in the Declaration of Helsinki. Written informed consent was obtained from all participants.

### Study site and study population

This was a cross sectional descriptive and analytic study among patients reporting at least one life time episode of UGIB, aged 12 years and above who presented at Pakwach Health Centre IV over the study period 14th July to 30th august 2014. Pakwach Health Centre IV is located in rural Uganda at banks of the Albert Nile River. The prevalence of schistosomiasis due to S. mansoni in this region is reported at over 50% and facility records report a relatively high frequency of patients with UGIB. Generally, 30 patients above the age of 5 years are seen at outpatients daily and about 4 to 5 adult patients, not including pregnant women are admitted every day. Data from the Uganda national health management information systems for the year 2012/13 recorded 24,222 outpatient visits and 5532 inpatient admissions.

### Sample size estimation

Sample size for this cross sectional study design was estimated at 102 participants (n) using a power (a) of 90%, p=15%, N= 285, d= 5% using the formula, n = [(N p (1-p)]/ [(d2/Z21-a/2*(N-1)+p*(1-p)]. Where p is the proportion of patients estimated to develop upper gastrointestinal bleeding due to varices, N is the number patients from this health facility meeting our inclusion criteria in a year, Z is the Z-score, and d is the confidence limits.

### Enrolment procedures and data collection

Screening and enrolment were carried during the working week. Patients receiving care at the outpatients and in patient departments at Pakwach health Centre IV were assessed for eligibility. The inclusion criteria comprised aged 12 years and above, written informed consent /assent, and reporting at least one lifetime episode of UGIB. Participants with any absolute or relative contraindication to un-sedated diagnostic upper gastrointestinal endoscopy were excluded from the study. These comprised pregnant women, patients requiring or under resuscitation, and those unable to tolerate the procedure. During screening we employed systematic sampling at outpatients (skipped the first three registered patients and approached every other patient aged 12 years and over) and for ethical reasons recruited all eligible inpatients with acute upper gastrointestinal bleeding (AUGIB) after resuscitation and stabilization during the study period since they would benefit from endoscopy. In the study, UGIB was defined as a lifetime of any of the following: hematemesis and melena or blood in stool as reported by the eligible study participant. However, the main outcome for this study was number of lifetime episodes of UGIB reported by a participant. We recorded socio-demographic data, exposure to schistosoma species or alcohol, treatment of schistosomiasis and time from the last treatment, history of UGIB, related past medical, or drug treatment history, stigmata of chronic liver disease, vital signs, 3-part hematology indices (using compact Sysmex KX-21 hematology analyzer), hepatitis B and C viral blood serology results (obtained by commercially available rapid diagnostic test kits), malaria antigen test results (by rapid diagnostic test kits), stool microscopy for ova, urine circulating cathodic antigen (CCA) test by Rapid diagnostics, and ultrasound findings performed by a trained sonographer according to the modified World Health Organization Niamey protocol. Trans-abdominal ultrasound was performed using the SONOSTAR model SS8, portable ultrasound with a 3.5 MHz convex probe. All participants were then scheduled for diagnostic upper digestive endoscopy that was performed using Pentax EPKi digital video processor and Pentax 9.8 mm video gastroscope for diagnosis. At the commencement of the procedure the patient was gowned, he or she was asked to sit on the procedure bed and xylocaine oral spray administered. He or she was then positioned in the left lateral position and a mouth gag inserted. The oesophagus, stomach, and duodenum were examined for evidence suggestive of UGIB or cause of UGIB with special emphasis on varices, oesophageal or gastric erosions or ulcers. Endoscopic findings were assessed and reported as recommended by the Japanese Research For Portal Hypertension [24] and /or the modified Forrest classification upper gastrointestinal bleeding [25] in the clinical report form.

### Data management and analysis

Data obtained from these study procedures was recorded into a clinical report form and transcribed into Microsoft Access database software 2007 (Microsoft corporation) on a daily basis. This was later edited to ensure quality and exported to Stata version 13 (STATACorp, Lakeway, College Station, Texas, USA). Within Stata, descriptive and inferential statistics were undertaken profiling the study population and factors associated with episodes of UGIB. Contingency tables summarizing the factor variables relative to the outcome were generated and inference determined by likelihood ratio Chi-square test. Further statistical inference was determined by negative binomial multivariate analysis of the count data with robust estimator of variance. A significance level (p-value < 0.05) was considered, confidence intervals supported inference, and beta coefficients provided a measure of how strongly each factor variable influenced the outcome variable. Selection of the best-fit models was criterion based. Results of the best-fit model (the model with the lowest Akaike information criterion-AIC, and Bayesian information criterion-BIC) were then subject to marginal analysis and which are summarized as a graph. These results are presented in the body of the manuscript, as tables, and as figures.

## Results

Over a period of 6 weeks (July 2014 to August 2014), one hundred and seven participants were enrolled. With exception of one participant that reported once a week contact with the waters of the Nile, all other reported 5-7 times a week contacts with the waters of the Nile. Twenty-three were in patients with acute severe UGIB, and 84 outpatients with a past history of UGIB. The prevalence of UGIB among screened at outpatients was 24%; 95%CI (20%-29%). The age range of participants was 25 to 71 years. Sixty percent were females and 96% reported ever experiencing hematemesis. The rest reported melena or hematochesia without hematemesis. The total number of episodes of UGIB reported in the study was 323 episodes. Sixty-one (57%) participants experienced two or more episodes of UGIB during their lifetime and the rest reported experiencing only one episode of UGIB during their lifetime. Seventy-five percent of the study participants experienced their first episode of UGIB within 5 years of enrollment. Ninety-six percent were ever admitted for UGIB, no participant had ever had an endoscopy for their UGIB and 73% previously received blood transfusion for UGIB. Severe chronic hepatic schistosomiasis was diagnosed with certainty in 101 (94%) participants, however alcohol use, serological markers of chronic viral hepatitis, and active infection with schistosoma mansoni were not that frequent. Notably, 78% of our study participants had received praziquantel for treatment of schistosomiasis in the last 12 months. With exception of splenomegaly that was found among 92(90%) participants, stigmata of chronic liver disease were not frequent in our study. More than 50% of our participants had anemia (hemoglobin level< 110g/L) and thrombocytopenia (platelet count < 150x109/L). Esophageal varices were the most common endoscopic finding explaining UGIB and these was found among 80% of our study participants (Table 1).

**Table 1:** Baseline characteristics of all participants

	N (%)
Age in years Median (Interquartile range)	45(13)
Female	64(60)
Hematemesis	103 (96)
Melena	47(44)
Hematochesia	55(51)
Admitted with severe acute UGIB	23(22)
Prevalence of UGIB among out patients	84(24)
Total Episodes of UGIB reported in the study	323
Episodes of UGIB reported by each participant Median (Interquartile range)	2 (3)
Previously or currently admitted for UGIB	103 (96)
Ever had an endoscopy for UGIB	0
Had a blood transfusion for UGIB	73(68)
Certain Hepatic schistosomiasis (Ultrasound patterns D, E, F, X and/ or previous ova of S. mansoni in stool)	101 (94)
Praziquantel last 12 months	70(65)
Alcohol use	4 (4)
Liver flap	10 (9)
Ascites on examination	18 (17)
Palpable spleen	92 (90)
Ascites on ultrasound	15 (14)
Splenomegaly on ultrasound (spleen diameter ≥12cm)	86 (82)
Portal vein diameter ≥1.3cm	75 (73)
Niamey liver ultrasound protocol No or mild periportal fibrosis (pattern A, C,) Moderate or Severe periportal fibrosis (pattern D, E, F)	9 (8) 59 (55) 39 (37)
Collaterals at ultrasound	5 (5)
Hepatitis B surface antigen positive	7 (7)
Hepatitis C antibody positive	28 (26)
Treated with praziquantel in the last 12 months	70 (78)
Schistosoma mansoni ova in stool smear	7 (7)
Positive urine circulating cathodic antigen (CCA) test	9 (8)
Hemoglobin level (g/L) Median (Interquartile range)	102 (45)
Platelet count (x109/L) Median (Interquartile range)	54 (53)
Esophageal varices at endoscopy Portal hypertensive gastropathy Peptic ulcer disease at endoscopy	86 (80) 83 (78) 18 (17)

UGIB-upper gastrointestinal bleeding

### Clinical factor variables by number of lifetime episodes of UGIB

The frequencies of lifetime episodes of UGIB among the 3 different categories of bleeding “1 episode”, “2 or 3 episodes”, “4 or more episodes”, were 46, 81, and 196 respectively. Participants who experienced more than one episode of UGIB were likely to have the following; aged =40 years, females, severe acute variceal bleeding, history of variceal bleeding, previous treatment with praziquantel, an enlarged spleen, ascites, abdominal collaterals, pattern X according to Niamey protocol, hemoglobin level =90g/L, platelet count = 35 x109/L cells, and esophageal varices at endoscopy. In contrast, participants with one episode of UGIB were likely to have a liver flap and a positive hepatitis B surface antigen test (Table 2).

**Table 2:** A contingency table summarizing different of episode categories of upper gastrointestinal bleeding and total number of episodes of upper gastrointestinal bleeding by various clinical factor variables

Categories of UGIB	1 episode of UGIB	2 or 3 episodes of UGIB	≥4 episodes of UGIB	
Number of participants per category	46	34	27	107
Total number of episodes of UGIB per category	46	81	196	323
	N (%)	N (%)	N (%)	P-value*
Age 40 years	32(70)	54(67)	177(90)	0.000
Female	25 (54)	50(62)	143(73)	0.025
Severe acute UGIB	5(11)	25(31)	71(36)	0.002
History of blood transfusion	20(44)	66(82)	192(98)	0.000
Active infection with Schistosoma mansoni	6(13)	5(6)	7(4)	0.068
Praziquantel in 12 months	28(80)	49(71)	153(80)	0.328
Liver flap	7(15)	4(5)	8(4)	0.037
Spleen diameter 12cm	35(76)	69(85)	179(91)	0.021
Ascites at ultrasound	7(15)	6(7)	51(26)	0.001
Collaterals at ultrasound	0	5(6)	19(10)	0.011
Niamey protocol Periportal ultrasound patterns A, C, D, E, F X	5(11) 27(59) 14(30)	8(10) 46(57) 27(33)	4(2) 89(45) 103(53)	0.001
Hemoglobin 90g/L	13(28)	35(43)	132(67)	0.000
Platelet count 35x10^9^/L	6(13)	20(25)	92(47)	0.000
Hepatitis B surface antigen Positive	5(11)	3(4)	4(2)	0.044
Esophageal varices at endoscopy	33(72)	64(79)	192(98)	0.028

UGIB- upper gastrointestinal bleeding.

* P-value generated from the likelihood ratio Chi square test.

### Multivariable analysis output for the three best-fit models

We identified 3-fit models. Model-3 performed best since it demonstrated the lowest Akaike information criterion and Bayesian information criterion. Model-3 (n=68) included all the factor variables listed and limited to patients who received praziquantel in the last one year. In contrast, model-1 (n=88) included one-year treatment with praziquantel as a factor variable and model-2 (n=105) included all participants not correcting for treatment with praziquantel (Table 3). A summary of the regression coefficients, their standard errors, constants, p-values, and criterion are presented (Table 3).

**Table 3:** A summary of the regression coefficients, their standard errors (parentheses), constants, p-values and criterion measures for the 3 best-fit models

	Model 1(N=88)	Model 2(N=105)	Model 3(N=68)
Age 40 years	0.607***	0.592***	0.780***
	(-0.178)	(-0.163)	(-0.208)
Female	0.493**	0.412*	0.784***
	(-0.162)	(-0.172)	(-0.175)
History of Blood transfusion	0.656***	0.707***	0.539**
	(-0.152)	(-0.129)	(-0.169)
Abdominal collaterals	0.472	0.515	0.841**
	(-0.374)	(-0.344)	(-0.276)
Esophageal varices	0.437**	0.408**	0.450*
	(-0.163)	(-0.137)	(-0.194)
Pattern-X periportal fibrosis	0.233	0.176	0.352*
	(-0.16)	(-0.173)	(-0.169)
Hemoglobin 90g/L	0.428**	0.393**	0.347*
	(-0.144)	(-0.14)	(-0.151)
Platelet count ≤35x109/L	0.478**	0.415*	0.359*
	(-0.175)	(-0.169)	(-0.16)
Praziquantel in 12 months	-0.115	-	-
	(-0.198)	-	-
Model constant	-0.902***	-0.930***	-1.269***
	(-0.272)	(-0.242)	(-0.314)
AIC	354.7	408	268.3
BIC	382	434.6	290.5

* p<0.05

** p<0.01

*** p<0.001

AIC -Akaike information criterion, BIC- Bayesian information criterion.

### Impact of factor variables on the number of lifetime episodes of UGIB for model-3

**Figure 1:**
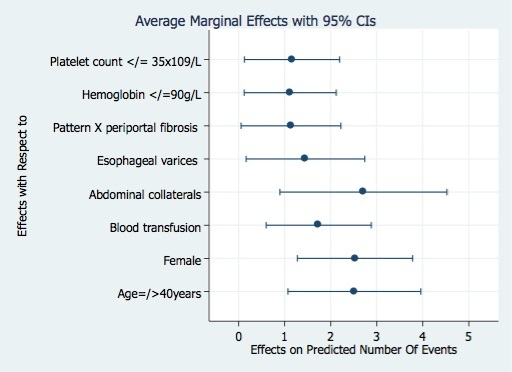
Average marginal effects with 95% confidence intervals of factor variable from model-3

Average marginal effects were calculated from model-3 and presented as a graph, describing the positive impact of the various factor variables on the number of lifetime episodes of UGIB reported by our study participants (Figure 1). On average the presence of any of these factors variables in a participant would increase the individuals probability of experiencing two or more l lifetime episodes of UGIB (Figure 1).

## Discussion

Upper gastrointestinal bleeding is poorly described at primary health care facilities in rural Africa because weak health systems [26–28]. We studied at episodes of upper gastrointestinal bleed as reported by the patients to describe upper gastrointestinal bleeding at a rural primary health care facility. We found upper gastrointestinal bleeding was a common health problem at this primary health facility in rural sub Saharan Africa most participants reported more than 2 lifetime episodes of UGIB. Patient reported upper gastrointestinal bleeding was corroborated by endoscopy, abdominal ultrasound, and presence of anemia [29–31]. Endoscopy and ultrasound showed most participants had esophageal varices and/or meet the ultrasound criteria diagnostic of hepatic schistosomiasis. Moreover, similar findings have been reported by hospital-based studies from the same region[8, 9, 13, 17, 32]. In contrast, there was limited evidence to suggest UGIB in our study was mainly due to cirrhosis due to hepatitis or peptic ulcer disease. The study also identified a cluster of factor variables, which were associated with an increased probability of experiencing two or more episodes of UGIB in this population. We found an association between age =40years and number of episodes of UGIB. Others showed individuals aged over 35 years with schistosomiasis had the highest frequency of periportal fibrosis [33] and were 2 times more likely to develop UGIB [34]. We also found an association between female gender and likelihood of experiencing increasing episodes of UGIB. One study reported females are more likely to present with UGIB due to varices than males [35]. However, we do not have any clear explanation for this peculiar finding. A history of blood transfusion was one of the other factors associated with increasing episodes of UGIB. A number of studies show blood transfusion is associated with recurrent episodes of UGIB [36, 37]. It could be argued that each bleeding episode was a trigger for blood transfusion and since our patients were able to survive this long, the association that we are observing is just an accumulation of several events. On the other hand, some clinical trials show restricting blood transfusion among patients with UGIB was shown to reduce UGIB recurrences and mortality [37, 38]. Periportal fibrosis, abdominal collaterals, esophageal varices, anemia, and thrombocytopenia are recognized causes or sequel of portal hypertension. All these factors have been associated with recurrent UGIB. Our findings are comparable with what has been reported by others [12, 20, 39–43]. The negative correlation of hepatitis B surface antigen or liver flap with increasing number of lifetime episodes of UGIB suggests patients with these factors may not have survived. This explanation is supported by two publications that indicate these two factors are associated with increased risk of death [44, 45]. Our study had limitations. These are partly related to its cross sectional study design and the fact participants were selected from a single primary health care facility. Moreover, our study might have been exposed to recall bias because we relied on anamnesis to retrospectively reconstitute the first hand past experience of our participants. However our clinical findings, ultrasound, laboratory, and endoscopic were in keeping with the participant’s account of UGIB [30, 46–49].

## Conclusion

Our study high lights upper gastrointestinal bleeding is a common health problem in rural SSA. In addition, the study provides an up-to-date profile of UGIB in rural SSA where schistosomiasis is endemic. These are important findings for populations at risk of hepatic schistosomiasis, for better case management, improvement of health management information systems, and further research in SSA.

### What is known about this topic

- Severe hepatic schistosomiasis and/or liver cirrhosis are the most common causes of upper gastrointestinal bleeding in sub Saharan Africa;
- Mass drug administration with praziquantel has been shown to reduce hepatic schistosomiasis and its complications in Africa;
- There is limited clinical epidemiological data on upper gastrointestinal bleeding especially from rural Africa where schistosomiasis is endemic.

### What this study adds

- UGIB is still a common health problem among communities were schistosomiasis is endemic despite mass drug treatment with praziquantel and mainly affects those within the age of 25 years to 70 years;
- The study provides up-to-date clinical epidemiological data that be used locally and regionally for development of disease management guidelines, improvement of health information management systems, and health resource planning;
- The study provides the premise for further clinical research among patients with UGIB related to hepatic schistosomiasis in SSA.
